# Optimizing sedation in gastroscopy: a study on the etomidate/propofol mixture ratio

**DOI:** 10.3389/fmed.2024.1392141

**Published:** 2024-06-12

**Authors:** Shuyi Tang, Yuling Zheng, Xiaoli Li, Yiwen Zhang, Zhongqi Zhang

**Affiliations:** ^1^Department of Anesthesiology, Shunde Hospital of Southern Medical University, Foshan, China; ^2^Department of Anesthesiology, The Affiliated Shunde Hospital of Jinan University, Foshan, China

**Keywords:** etomidate-propofol mixture, gastroscopy, hemodynamics, sedation, adverse reactions

## Abstract

**Objective:**

Propofol and etomidate are the most commonly used sedative agents in procedural sedation, each with its own advantages and disadvantages. However, there remains considerable controversy regarding the optimal ratio for the mixture of these two drugs, warranting further investigation. Therefore, this study aims to investigate the optimal ratio for combining propofol and etomidate during gastroscopy.

**Methods:**

This study is a prospective, double-blinded, randomized controlled clinical trial. One hundred and sixty-two patients from July 2019 to December 2022 were evenly classified into three groups using a random number table as follows: (1) P group (propofol); (2) EP1 group (5 mL etomidate +10 mL propofol); (3) EP2 group (10 mL etomidate +10 mL), 54 patients per group. The medications, including a pre-sedation dose of 50 μg/kg dezocine followed by sedatives, ceasing when the patient’s eyelash reflex vanished, indicating adequate sedation. Mean arterial pressure (MAP), heart rate (HR), and peripheral oxygen saturation (SpO_2_) measurements taken before anesthesia (T1), immediately after the administration of sedatives (T2), immediately gastroscopic insertion (T3) and immediately recovery (T4) were determined. Additional, perioperative related outcomes and adverse events were also recorded.

**Results:**

The EP2 group exhibited a higher MAP at T2 compared to the P and EP1 groups (*p* < 0.05). Calculated decreases in MAP revealed values of 19.1, 18.8, and 13.8% for the P, EP1, and EP2 groups at T2, respectively. Adverse events: Group EP2 exhibited a significantly lower hypotension incidence (11.1%) compared to the Propofol group (50%) and EP1 (31.5%). Concerning injection pain, Group EP2 also showing a significant decrease in comparison to P and EP1 groups (*p* < 0.05).

**Conclusion:**

The use of a mixture of 10 mL etomidate and 10 mL propofol (at a 1:1 ratio) combined with dezocine for painless gastroscopy demonstrates hemodynamic stability, a low incidence of adverse reactions.

**Clinical Trial Registration:**

https://www.chictr.org.cn/showproj.html?proj=39874

## 1 Introduction

Gastroscopy is an invasive procedure, generally performed under procedural sedation to ensure patient comfort. The protocol utilized aims to achieve rapid induction, precise effect, stable hemodynamics, rapid recovery, and minimal adverse reactions (1–3). Patients undergoing gastroscopy often experience complications related to the cardiovascular or respiratory systems, making it challenging to perform the procedure safely and effectively while maintaining stable hemodynamics and minimizing respiratory depression (4). Hence, the selection of appropriate sedatives is crucial.

Propofol stands out as one of the most commonly used sedatives in various endoscopic procedures due to its high lipid solubility, rapid crossing of the blood–brain barrier, quick onset of action, and short recovery period (5, 6). A multicenter study revealed that approximately 30% of gastrointestinal endoscopies were conducted using propofol (7). However, respiratory and circulatory depression and injection pain represent the most common side effects of propofol, significantly limiting its usage. Etomidate, another widely used sedative in clinical practice, shares similar properties with propofol and finds extensive application in gastroscopy (8). It is a short-acting intravenous drug that offers distinct advantages in hemodynamics and respiration compared to propofol, with minimal impact on heart rate and blood pressure, rendering it safe for use in patients with unstable cardiovascular systems (9). Its rapid onset, short recovery time, stable hemodynamics, and mild respiratory depression provide reliable procedural sedation conditions for comfortable medical procedures (10). Although etomidate has a lower incidence of respiratory arrest and hypoxemia, as well as reduced injection pain, and does not increase the risk of postoperative nausea and vomiting, it does exhibit a higher occurrence of myoclonus and postoperative nausea and vomiting (11).

Observations indicate that the pharmacological characteristics of etomidate and propofol complement each other, and researchers have tried to highlight the benefits of both drugs and reduce the adverse reactions produced by single drug use by using a combination of etomidate and propofol. Nevertheless, considerable controversy surrounds the optimal ratio of the two drugs in the mixture, leading to potential variations in sedative effects and the incidence of adverse reactions such as hypotension, respiratory depression, myoclonus, and postoperative nausea and vomiting. Consequently, this study employed an etomidate/propofol mixture for sedation during gastroscopy to investigate the appropriate ratio of the two drugs.

## 2 Materials and methods

### 2.1 Study design

This study is a prospective, double-blinded, randomized controlled clinical trial, authorized by the hospital ethics committee (approval number: 20190576), registered at the China Clinical Trial Registry http://www.chictr.org.cn/ (registration number: ChiCTR1900023875). All included patients or their family members signed informed consent forms. Patients who voluntarily underwent painless electronic gastroscopy examination from July 2019 to December 2022 were selected.

### 2.2 Inclusion and exclusion criteria

#### 2.2.1 Inclusion criteria

Eligible participants are adults aged 18 to 65, undergoing elective or diagnostic gastroscopy, classified as American Society of Anesthesiologists (ASA) class I or II, and with a body mass index (BMI) ranging from 17.5 to 27 kg/m^2^.

#### 2.2.2 Exclusion criteria

Participants will be excluded if they refuse participation, exhibit severe hepatic or renal dysfunction, suffer from chronic pain or mental disorders, have symptomatic cardiovascular or pulmonary conditions, experience drug allergies related to the study medications, have obstructive sleep apnea-hypopnea syndrome, require hemostasis, polypectomy, or other interventions before or during the procedure, have a history of alcoholism or recent use of psychotropic medications, or have taken analgesics within 24 h preceding the gastroscopy.

### 2.3 Randomization and blinding

Patients were classified into three groups using a random number table as follows: (1)P group (propofol); (2)EP1 group (5 mL etomidate +10 mL propofol); (3)EP2 group (10 mL etomidate + 10 mL propofol).

Randomization of participants was facilitated by a computer-generated system, with the resulting assignment information securely enclosed in opaque envelopes, the patients were blinded to group allocation. All gastroscopy procedures were conducted by skilled endoscopists boasting over 5 years of experience. An independent observer, blind to the participants’ group assignments, was responsible for the collection and documentation of pertinent data. Given that propofol and etomidate are visually indistinguishable, each was prepared and then drawn into a standardized 20 mL syringe up to 15 mL by a separate researcher not involved in the procedure. This ensured that both the administering physician and the observer remained unaware of the specific sedatives used. A specialized statistician was tasked with the statistical analysis of the gathered data.

### 2.4 Anesthesia procedure

In this study, all participants received a standardized anesthesia protocol. The medications, including a pre-sedation dose of 50 μg/kg dezocine followed by sedatives (either propofol or a mixture of etomidate/propofol), were prepared by designated research personnel. Etomidate, sourced from Zhejiang Jiuxu Pharmaceutical Co., Ltd. (2 mg/mL, 10 mL, batch number: YT201026), and Propofol, obtained from Fresenius Kabi (20 mg/mL, 20 mL, batch number: 16NM6293), were administered at least 5 min after dezocine. The intravenous administration was timed to last no less than 60 s, ceasing when the patient’s eyelash reflexes vanished, indicating adequate sedation. During the gastroscopy, any patient movement, coughing, or factors impeding the procedure warranted an additional 2–5 mL of the sedative, with further doses as necessary. The target sedation level was achieved when the patient’s eyelash reflex disappeared, followed by a gastroscopy performed by an endoscopist. Post-gastroscopy, patients were monitored in the post-anesthesia care unit (PACU) until their vital signs stabilized and no significant adverse events were observed, marking their readiness for discharge.

### 2.5 Sample size estimation

The primary outcome measure for this study was the mean arterial pressure (MAP) following sedative administration. We established two groups in the pre-trial: a control group (administered propofol) and an experimental group (given mixture of 5 mL etomidate + 10 mL propofol), with 10 patients in each. Preliminary results indicated a MAP of 75.2 ± 6.5 mmHg after administration of propofol alone, compared to 80.1 ± 8.2 mmHg following the combined administration of propofol and etomidate mixture. With a significance level (α) set at 0.05 and a power (β) of 0.9, accounting for a potential data missing or loss rate of 10%, the calculated sample size required for each group was 54 subjects. Therefore, the total sample size necessary for the study was 162 subjects.

### 2.6 Outcomes

1. Primary outcomes: The MAP immediately after intravenous injection of sedative medication.
2. Additional outcomes included MAP, heart rate (HR), and peripheral oxygen saturation (SpO_2_) measurements taken before anesthesia (T1), immediately after the administration of sedatives (T2), immediately gastroscopic insertion (T3) and immediately recovery (T4). Gastroscopy time, patient waking time, total dosage of sedatives used, along with the satisfaction levels of both the endoscopic physician and the patient, which were classified as very satisfied, satisfied, or dissatisfied. Furthermore, occurrences of hypotension, hypertension, bradycardia, tachycardia, hypoxemia, injection pain, muscle spasm, postoperative nausea and vomiting (PONV), dizziness, and postoperative abdominal pain were recorded.

Recovery time was defined as the duration from the last sedative administration until the patient regained consciousness and could respond independently. Hypoxemia was defined as SpO_2_ levels falling below 90% during the examination, prompting interventions such as jaw support, airway opening, and mask-assisted oxygen administration. Bradycardia was identified as HR < 50 bpm, and treated with 0.3–0.5 mg atropine. Hypotension (a MAP decreases of >20% from baseline on two consecutive readings) was managed with 5–10 mg ephedrine. PONV was managed with intravenous tropisetron 2 mg.

### 2.7 Statistical analysis

Statistical analyses were conducted using IBM SPSS Statistics version 22.0. Continuous variables are presented as means ± standard deviation (SD). The normality of data distribution was assessed using the Kolmogorov–Smirnov test and visual inspection of histograms. For comparisons among the three groups, one-way Analysis of Variance (ANOVA) was employed. Categorical data comparisons were performed using the Chi-square test or Fisher’s exact test, as appropriate. Repeated measures ANOVA (RANOVA) was utilized to analyze vital sign measurements taken at various time points within each group. The Bonferroni correction method was employed to address correction for multiple comparisons. A *p*-value of less than 0.05 was considered indicative of statistical significance.

## 3 Results

### 3.1 General information comparison of patients

**Figure 1:**
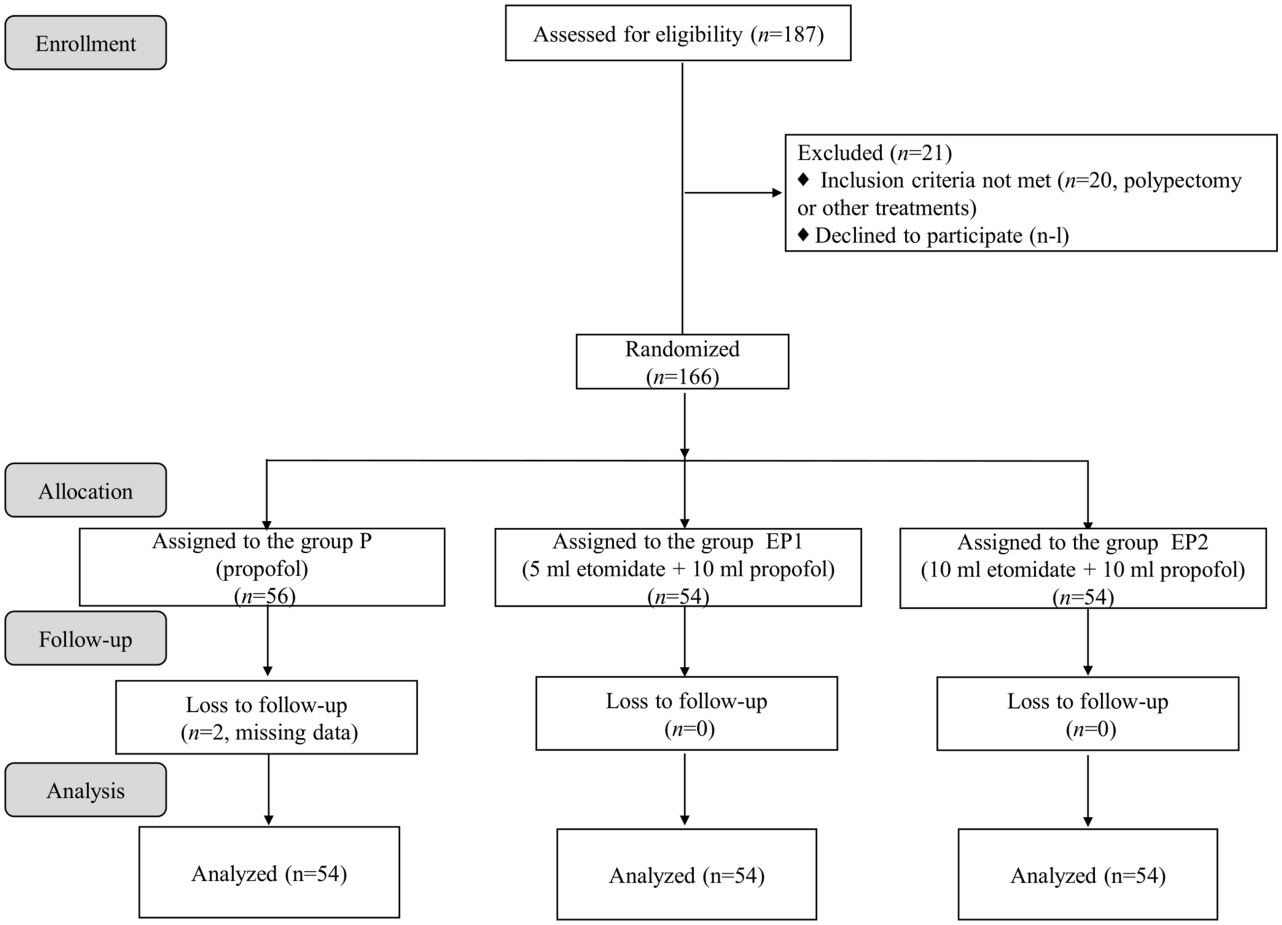
Flow diagram of the study.

A total of 187 patients were initially enrolled in the study. Of these, 21 patients were excluded for not meeting the inclusion criteria, leaving 166 patients to be randomly allocated into three groups: P group (*n* = 56), EP1 group (*n* = 54), and EP2 group (*n* = 54). Due to 2 instances of data attrition in the P group, the final analysis was conducted with 162 patients, distributing 54 participants equally across each group, as depicted in Figure 1. The baseline characteristics of patients across the three groups including gender, age, height, weight, BMI, ASA, and histories of smoking, drinking, hypertension, and diabetes were compared and found to have no statistically significant differences (*p* > 0.05), as summarized in Table 1.

**Table 1:** Characteristics of the patients in three groups.

Index	P group	EP1 group	EP2 group	*p*-value
Gender (male/female)	24/30	29/25	20/34	0.218
Age (years)	44.9 ± 12.2	46.6 ± 10.7	48.3 ± 10.2	0.271
Height (cm)	163.8 ± 6.1	163.8 ± 6.5	161.7 ± 6.2	0.138
Weight (kg)	60.6 ± 8.4	60.2 ± 9.8	59.6 ± 8.2	0.834
BMI (kg/m^2^)	22.5 ± 2.0	22.3 ± 2.6	22.7 ± 2.4	0.668
ASA (I/II)	30/24	26/28	27/27	0.725
History of smoking n (%)	8(14.8)	12(22.2)	9(16.7)	0.579
History of drinking n (%)	10(18.5)	8(14.8)	8(14.8)	0.833
History of hypertension n (%)	8(14.8)	10(18.5)	10(18.5)	0.841
History of diabetes n (%)	10(18.5)	12(22.2)	14(25.9)	0.651

Data are mean ± SD or *n* (%). ANOVA, Chi-square test or Fisher’s exact test was utilized to assess differences.

### 3.2 Changes in hemodynamics at different time points

Following the intravenous administration of sedatives, all patient groups experienced a significant reduction in MAP at the T2 and T3 time points compared to baseline (*p* < 0.05). Notably, the EP2 group exhibited a higher MAP at T2 compared to the P and EP1 groups (*p* < 0.05). Calculated decreases in MAP revealed values of 19.1, 18.8, and 13.8% for the P, EP1, and EP2 groups, respectively. Furthermore, significant decreases in HR were observed across all groups post-sedation, with statistical differences compared to baseline (*p* < 0.05). HR levels tended toward normalization upon awakening. Regarding SpO_2_, all groups exhibited a significant decrease at the T2 and T3 time points compared to baseline (*p* < 0.05). However, the decrease was more pronounced in the P group. Notably, at the T2 time point, both EP1 and EP2 groups showed higher SpO_2_ levels compared to the P group (*p* < 0.05). At time points T2 and T3, there were significant differences among the three groups in terms of MAP, HR, and SpO_2_ (*p* < 0.05). The three hemodynamic parameters of MAP, HR, and SpO2 all varied significantly over time (*p* < 0.001), exhibited a trend of initial decline followed by increase, as shown in Table 2.

**Table 2:** Comparison of hemodynamics at different time points among three groups.

Index	Time point	P group	EP1 group	EP2 group	*P*-value
MAP (mmHg)	T1	93.7 ± 8.3	93.1 ± 8.9	95.1 ± 10.3	0.465
	T2	75.1 ± 5.3^c^	75.6 ± 6.4^c^	82.0 ± 8.3^abc^	<0.001
	T3	79.3 ± 6.5^c^	80.4 ± 5.7^c^	84.2 ± 7.7^abc^	<0.001
	T4	90.5 ± 7.7	89.4 ± 8.7	93.1 ± 9.6	0.187
		*P* < 0.001	*P* < 0.001	*P* < 0.001	
HR (beat/min)	T1	74.1 ± 6.3	74.6 ± 7.1	73.7 ± 6.9	0.238
	T2	65.2 ± 6.4^c^	63.9 ± 8.3^c^	65.1 ± 8.7^c^	0.026
	T3	68.5 ± 6.6^c^	69.2 ± 7.3^c^	69.3 ± 7.7^c^	0.001
	T4	76.9 ± 7.3	73.6 ± 6.9	74.2 ± 6.6	0.193
		*P* < 0.001	*P* < 0.001	*p* < 0.001	
SpO_2_%	T1	99.5 ± 0.6	99.6 ± 0.6	99.5 ± 0.6	0.516
	T2	96.5 ± 4.2 ^c^	98.3 ± 2.2^ac^	98.4 ± 2.5^ac^	0.002
	T3	98.3 ± 2.6 ^c^	99.3 ± 1.2^a^	99.2 ± 1.0^a^	0.004
	T4	99.4 ± 0.8	99.7 ± 0.4	99.5 ± 0.6	0.313
		*P* < 0.001	*P* < 0.001	*p* < 0.001	

Data are mean ± SD. Compared with group P at the same time point, ^a^*P* < 0.05; compared with group EP1 at the same time point, ^b^*P* < 0.05; compared with T1 within the group, ^c^*P* < 0.05. MAP, mean arterial pressure; HR, heart rate; SpO_2_, peripheral oxygen saturation. ANOVA or RANOVA was utilized to assess differences.

### 3.3 Perioperative related outcomes

Significant variations were observed in the total sedative dosage among the three groups (*p* = 0.024). Specifically, the EP1 and EP2 groups received significantly higher sedative dosages compared to the P group (*p* < 0.05). In contrast, the analysis revealed no significant differences in the examination time, recovery time, satisfaction of endoscopists and patient satisfaction across all groups (*p* > 0.05), as presented in Table 3.

**Table 3:** Comparison of perioperative related outcomes among three groups.

Index	P group	EP1 group	EP2 group	*P* value
Total sedative dosage (mL)	16.4 ± 3.3	17.8 ± 3.9^a^	18.1 ± 2.8^a^	0.024
Examination time (min)	5.9 ± 0.7	6.2 ± 0.8	6.2 ± 0.6	0.139
Recovery time (min)	11.7 ± 1.7	11.9 ± 1.8	12.3 ± 1.5	0.174
Endoscopist satisfaction (*n*) (very satisfied/satisfied/dissatisfied)	47/6/1	45/7/2	42/8/4	0.680
Patient satisfaction (*n*) (very satisfied/satisfied/dissatisfied)	49/5/0/	48/6/0	47/6/1	0.950

Data are mean ± SD or *n*. Compared with group P, ^a^*P* < 0.05. ANOVA, Chi-square test or Fisher’s exact test was utilized to assess differences.

### 3.4 Adverse events

Substantial differences were observed in hypotension rates among the three groups (*p* < 0.001); Group EP2 exhibited a significantly lower hypotension incidence (11.1%) compared to the P group (50%) and EP1 (31.5%). In terms of hypoxemia, identified as SpO_2_ levels falling below 90% during the perioperative period, notable variations were recorded: 18.5% in the P group, and 3.7% in both EP1 and EP2 groups (*p* = 0.015); The incidence rates in EP1 and EP2 groups were significantly lower than in the P group (*p* < 0.05). Concerning injection pain, pronounced differences were evident among the groups (*p* < 0.001); The P group had the highest occurrence rate at 55.5%, followed by EP1 group at 27.8%, and EP2 group at a markedly lower 5.5%. Both EP1 and EP2 groups demonstrated significantly reduced pain incidence compared to the P group (*p* < 0.05), with EP2 group also showing a significant decrease in comparison to EP1 group (*p* < 0.05).

Other adverse reactions did not exhibit significant differences across the groups (*p* > 0.05), as detailed in Table 4.

**Table 4:** Comparison of adverse events between the three groups.

adverse events	P group	EP1 group	EP2 group	95% CI (EP2 vs. P, EP2 vs. EP1)	*P*-value
Hypotension	27(50)	17(31.5)	6(11.1)^ab^	(0.100, 0.495)	<0.001
				(0.151, 0.826)	
Hypertension	0(0)	0(0)	0(0)		-
Bradycardia	3(3.7)	1(1.8)	1(1.8)		0.620
Tachycardia	1(1.8)	0(0)	0(0)		0.673
Hypoxemia	10(18.5)	2(3.7)^a^	2(3.7)^a^	(0.046, 0.870)	0.015
				(0.146, 6.844)	
Injection pain	30(55.5)	15(27.8)^a^	3 (5.5)^ab^	(0.032, 0.308)	<0.001
				(0.061, 0.651)	
Mild	26	14	3		
Moderate	4	1	0		
Severe	0	0	0		
Muscle spasm	2(3.7)	5(9.3)	4(7.4)		0.628
PONV	7(12.9)	6(11.1)	4(7.4)		0.631
Dizziness	6(11.1)	4(7.4)	3(5.5)		0.673
Postoperative abdominal pain	1(1.8)	1(1.8)	0(0)		-

Data are *n* (%) or *n*. Compared with group P, ^a^*P* < 0.05; compared with group EP1, ^b^*P* < 0.05. Chi-square test or Fisher’s exact test was utilized to assess differences. CI, Confidence Interval.

## 4 Discussion

With the increasing demand for gastroscopy examinations due to the widespread use of gastrointestinal endoscopy, ensuring patient comfort while addressing new challenges for medical professionals becomes paramount. Anesthetic drugs play a pivotal role in achieving this balance. The study suggests that the use of an etomidate/propofol mixture (especially EP2 group, 10 mL propofol + 10 mL etomidate) could result in better hemodynamic stability during gastroscopy compared to propofol alone. This could be particularly beneficial for patients with cardiovascular concerns or those at higher risk of hemodynamic instability.

Propofol stands out as the primary agent in painless gastroscopy procedures due to its rapid onset, short duration of action, and swift recovery, along with its ability to mitigate postoperative nausea and vomiting (12). However, its respiratory depressant effects may lead to severe hypoxemia post-injection in susceptible patients (13). Furthermore, propofol’s potential to induce significant hypotension poses risks of compromised organ perfusion, increasing the likelihood of cardiovascular incidents (14). Additionally, propofol may cause injection pain, which may be related to direct stimulation of blood vessels or indirect stimulation of bradykinin and prostaglandin production (15, 16). It may even lead to serious complications such as acute refractory bradycardia leading to arrest (17). In our investigation, the incidence of hypotension following propofol administration alone reached 50%, with 55.5% reporting injection pain, and a proportion of low SpO_2_ below 90% as high as 18.5%. Etomidate acts primarily by enhancing the activity of the neurotransmitter GABA (gamma-aminobutyric acid) at its receptor in the central nervous system (18) and maintains more stable hemodynamics compared to many other sedatives, such as propofol. This characteristic renders etomidate particularly suitable for elderly patients with unstable circulation (19, 20) and exhibits a significantly lower incidence of injection pain compared to propofol (21). Despite these advantages, sole administration of etomidate can precipitate muscle spasms, postoperative nausea and vomiting, muscle pain, and potentially diminish adrenal function among other adverse reactions (22). Therefore, relying solely on etomidate for medical procedural sedation aimed at comfort might not be optimal. Exploring combination regimens that include etomidate plus other drug could offer a more balanced approach to procedural sedation, reducing adverse effects while enhancing patient comfort and safety.

Propofol also enhances GABAergic neurotransmission, thereby producing sedative effects (23, 24). Due to its pharmacological properties, the combination of propofol and etomidate presents a suitable solution for optimizing sedation during gastroscopy procedures. The synergistic effect of both drugs has been shown to mitigate adverse reactions associated with the individual application of propofol or etomidate, rendering the combination not only safer but also more effective (25, 26). In our study, we explored different volume ratios in the etomidate/propofol combination, including a 1:2 ratio (EP1 group) and a 1:1 ratio (EP2 group). The results demonstrated a noteworthy reduction in hemodynamic changes, muscle spasms, and injection pain compared to the separate use of propofol. Interestingly, the percentage decrease in mean arterial pressure (MAP) was more pronounced in the propofol group (P group) at 19.1%, compared to 18.8% in the EP1 group and 13.8% in the EP2 group. The incidence of hypotension followed a similar trend, with 50% in the P group, 31.5% in the EP1 group, and 11.1% in the EP2 group. From a hemodynamic perspective, our findings suggest that a higher proportion of etomidate contributes to more stable hemodynamics and a lower incidence of hypotension. Respiratory outcomes also favored the combination approach, as all three groups exhibited a decrease in SpO2, with the most significant decrease observed in the propofol group. At the T2 time point, the SpO2 levels in the EP1 and EP2 groups were higher than those in the P group (*p* < 0.05), and the incidence of hypoxemia in the EP1 and EP2 groups was significantly lower (18.5% vs. 3.7% vs. 3.7%). These results support the notion that combining propofol and etomidate of the 1:1 ratio in achieving stable hemodynamics and minimizing hypotension during gastroscopy procedures. However, there are also studies with different views. Lina et al. (27) reported a lower incidence of adverse reactions with a 1:2 volume ratio compared to the 1:1 ratio. Of course, the choice of etomidate/propofol ratio should consider factors such as patient characteristics, procedural complexity, treatment goals, drug dosage, and administration speed, these factors influence the outcome of the study (28–30). Patients with comorbidities or increased sensitivity to sedatives may benefit from lower etomidate doses to minimize hemodynamic instability (31, 32). Conversely, in lengthy or challenging procedures, a higher propofol concentration may be warranted to ensure adequate sedation depth.

Propofol is the main factor causing injection pain. Despite the administration of dezocine at a dose of 50ug/kg before administration of the sedatives, the P group exhibited a substantial incidence of injection pain at 55.5%, while the EP1 and EP2 groups showed significantly lower rates at 27.8% and 5.5%, respectively. Notably, the EP2 group demonstrated a notable reduction in injection pain incidence, suggesting a correlation with the etomidate mixture ratio. Higher etomidate ratios were associated with lower injection pain rates, potentially due to etomidate’s ability to diminish vascular intima stimulation (33). Additionally, our study revealed that although the P group had a higher proportion of injection pain, it mainly presented as mild pain [with a Visual Analog Scale (VAS) score of 1–3], not requiring any specific intervention.

This study has some limitations. Firstly, we have chosen the ASA I/II classification and an age range of 18–65 years, encompassing a wide demographic. The exclusion of high-risk populations may limit the generalizability of our findings, as different populations may exhibit varied responses to propofol/etomidate. Our primary focus on monitoring MAP changes after sedative administration may impact the adequacy of the sample size assessment. we recognize that the limitation in sample size may have affected the generalizability and reliability of our results. In future studies, we plan to increase the sample size of different populations to ensure a more representative dataset, thereby enhancing the credibility of our research findings. Second, the study details the incidence of adverse events such as hypotension and hypoxemia but does not fully explore all potential adverse reactions that could be associated with the sedative protocols used. A more comprehensive analysis of adverse events would provide a fuller safety profile of the sedative regimens. Third, the study only investigated a limited range of two ratios of etomidate/propofol, while there are infinite possible ratios. Other ratios may produce different effects, so future research could consider expanding the ratio range to comprehensively evaluate the effects of this drug combination. Therefore, further research is necessary to address these limitations.

## 5 Conclusion

The use of a mixture of 10 mL etomidate and 10 mL propofol (at a 1:1 ratio) combined with dezocine for painless gastroscopy demonstrates hemodynamic stability, a low incidence of adverse reactions. These findings suggest its potential for clinical application and anesthesiologists should consider incorporating the 1:1 etomidate-propofol combination into their sedation protocols during gastrointestinal endoscopy, especially for patients who may be at a higher risk of hypotension or other adverse effects when using propofol alone.

## Data availability statement

The raw data supporting the conclusions of this article will be made available by the authors, without undue reservation.

## Ethics statement

The studies involving humans were approved by Medical Ethics Committee of the Shunde Hospital of Southern Medical University. The studies were conducted in accordance with the local legislation and institutional requirements. The participants provided their written informed consent to participate in this study.

## Author contributions

ST: Writing – original draft. YuZ: Data curation, Writing – review & editing. XL: Writing – review & editing, Methodology. YiZ: Writing – review & editing, Data curation, Investigation. ZZ: Writing – review & editing, Conceptualization.
